# Yeast mitochondrial glutathione is an essential antioxidant with mitochondrial thioredoxin providing a back-up system

**DOI:** 10.1016/j.freeradbiomed.2016.02.015

**Published:** 2016-05

**Authors:** Irina Gostimskaya, Chris M. Grant

**Affiliations:** University of Manchester, Faculty of Life Sciences, The Michael Smith Building, Oxford Road, Manchester M13 9PT, UK

**Keywords:** AMS, 4-acetamido-4′maleimidyldystilbene-2,2′-disulphonic acid, CLS, chronological life span, Cys, cysteine, dNTP, deoxynucleotide, ER, endoplasmic reticulum, Glr1, glutathione reductase 1, GSH, reduced glutathione, GSSG, oxidized glutathione, Gpx, glutathione reductase 1, glutathione peroxidases, Fe–S, iron–sulphur, MetO, methionine sulphoxide, OD_600_, optical density at 600 nm, ONPG, o-nitrophenyl-β-D-galactopyranoside, OxD, degree of probe oxidation, ox, oxidized, ROS, reactive oxygen species, red, reduced, roGFP2, redox-responsive fluorescent protein, Prx, peroxiredoxin, SD, standard deviation, Trx, thioredoxin, Glutathione, Glutathione reductase, Mitochondria, Thioredoxin, Yeast

## Abstract

Glutathione is an abundant, low-molecular-weight tripeptide whose biological importance is dependent upon its redox-active free sulphydryl moiety. Its role as the main determinant of thiol-redox control has been challenged such that it has been proposed to play a crucial role in iron–sulphur clusters maturation, and only a minor role in thiol redox regulation, predominantly as a back-up system for the cytoplasmic thioredoxin system. Here, we have tested the importance of mitochondrial glutathione in thiol-redox regulation. Glutathione reductase (Glr1) is an oxidoreductase which converts oxidized glutathione to its reduced form. Yeast Glr1 localizes to both the cytosol and mitochondria and we have used a Glr1^M1L^ mutant that is constitutively localized to the cytosol to test the requirement for mitochondrial Glr1. We show that the loss of mitochondrial Glr1 specifically accounts for oxidant sensitivity of a *glr1* mutant. Loss of mitochondrial Glr1 does not influence iron–sulphur cluster maturation and we have used targeted roGFP2 fluorescent probes to show that oxidant sensitivity is linked to an altered redox environment. Our data indicate mitochondrial glutathione is crucial for mitochondrial thiol-redox regulation, and the mitochondrial thioredoxin system provides a back-up system, but cannot bear the redox load of the mitochondria on its own.

## Highlights

• The mitochondrial glutathione redox state, but not the cytoplasmic glutathione redox state, is crucial for oxidant tolerance.
• The mitochondrial thioredoxin acts as a back-up antioxidant system for the glutathione system.
• Mitochondrial glutathione and thioredoxin play an overlapping role in redox regulation which is important during ageing and oxidative stress conditions.

## Graphical abstract

**Figure:**
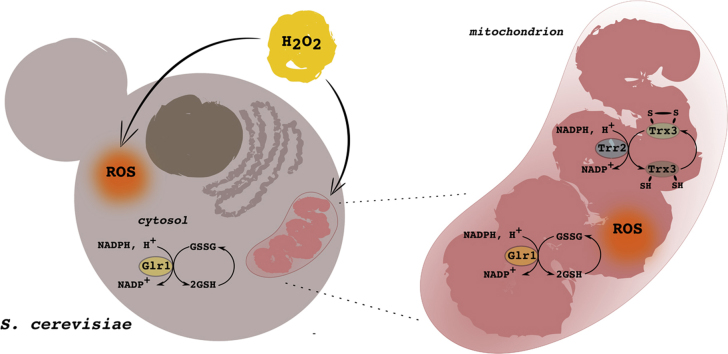

## 1 Introduction

Glutathione (GSH) is an abundant, ubiquitous low-molecular-weight tripeptide (γ-glutamylcysteinylglycine) whose biological importance is dependent upon its redox-active free sulphydryl moiety. It is an essential metabolite in eukaryotes, and for example, mice deficient in glutathione biosynthesis die rapidly [1]. Similarly, drug-induced glutathione depletion results in many tissue pathologies including haemolysis and defective brain function, cataract formation and oxidative damage to renal, hepatic and brain tissues [2]. Glutathione is synthesized in the cytosol of eukaryotic cells via two conserved, ATP-dependent steps, and is present intracellularly at concentrations up to 10 mM [3]. Its low redox potential (−240 mV) explains why it has long been considered the major cellular redox buffer and the redox state of the GSSG:2GSH redox couple is often taken as an indicator of the cellular redox environment [4]. However, this view of glutathione as the main determinant of thiol-redox control is increasingly being challenged, such that glutathione has been proposed to play only a minor role in thiol redox regulation, predominantly as a back-up system for the cytoplasmic thioredoxin system [5].

Much progress has been made towards understanding the eukaryotic requirement for GSH using the yeast *Saccharomyces cerevisiae* as a model organism. Initial studies confirmed that GSH is an essential metabolite during normal non-stress conditions [6], [7]. Mutants lacking *GSH1,* encoding the first step in GSH biosynthesis, are inviable, but viability can be maintained by the addition of exogenous GSH. Importantly, *gsh1* mutants undergo a limited number of cell divisions in the absence of GSH during which they utilize their pre-accumulated stores of GSH and hence the consequences of GSH depletion can be readily followed [8], [9]. Loss of GSH results in sensitivity to oxidative stress conditions, however, this requirement for GSH does not appear to depend on a role in ROS detoxification since the lethality of a *gsh1* mutant cannot be restored by growth under anaerobic conditions [9]. This raises the question as to the essential requirement for GSH. GSH is known to provide reducing power in dNTP synthesis with ribonucleotide reductase, although this is primarily thought of as a minor role compared with that of the thioredoxin system [10]. Mutants lacking *GSH1* also accumulate mitochondrial iron, which is a common phenotype in mutants that are defective in the maturation of cytosolic iron–sulphur (Fe–S) proteins [11], [12]. The finding that glutathione depletion results in an iron-starvation response and defects in cytosolic iron–sulphur proteins, without any apparent impact on cytosolic or endoplasmic reticulum (ER) thiol-redox control, was taken as evidence that the essential function of GSH is linked to iron metabolism, rather than thiol regulation [5], [13].

The essential requirement for GSH, together with the pleiotropic phenotypes which are observed in mutants depleted of GSH, particularly defects in iron metabolism, has meant that it has been difficult to define the role of GSH as an antioxidant. Whilst *gsh1* mutants depleted of GSH are sensitive to oxidants [6], [14], it is not clear what causes this sensitivity. In mammalian cells, glutathione peroxidases (Gpx’s) are thought to provide antioxidant activity. They do so by reducing a range of hydroperoxides to their corresponding alcohols using reducing power provided by GSH [15]. However, no equivalent Gpx enzymes have been described in yeast and the yeast Gpx-like enzymes (Gpx1-3) depend on the thioredoxin system for reduction [16], [17], [18]. One possibility is that GSH is required as an antioxidant in an organelle such as the mitochondria. This seems likely since mitochondria are a major source of cellular ROS [19] and maintain separate redox environments to the cytosol [20]. However, it is not currently possible to specifically deplete mitochondrial GSH, making it difficult to determine the mitochondrial requirement for GSH. In this current study we have taken the approach of utilizing a mutant lacking mitochondrial glutathione reductase, to examine the requirement for GSH in mitochondrial thiol-redox regulation.

Glutathione reductase (Glr1) is an NADPH-dependent oxidoreductase which converts oxidized GSSG to reduced GSH using reducing power generated by the pentose phosphate pathway. Yeast Glr1 is not essential for normal aerobic growth, but is required for viability during exposure to oxidative stress conditions [21]. Yeast Glr1 localizes to both the cytosol and mitochondria [22]. This localization is controlled by alternative start site selection at two in-frame AUG codons, in a mechanism that appears to be conserved for glutathione reductase in mammalian cells. Translation from the first AUG codon results in a long isoform containing a mitochondrial targeting sequence, whereas, translation from the second AUG codon results in a cytosolic form lacking the targeting sequence. This means that mutation of the first AUG codon (M1L mutant) results in an isoform of Glr1 which lacks the mitochondrial targeting sequence and Glr1 constitutively localizes to the cytosol [22]. We have used the Glr1^M1L^ mutant to examine the requirement for mitochondrial GSH. We show that it is the mitochondrial form of Glr1, rather than the cytosolic form, which is required for oxidative stress tolerance. Our data indicate that the GSSG:2GSH redox couple plays an essential function as a mitochondrial antioxidant, with the mitochondrial thioredoxin system providing only a minor back-up role. Our findings indicate that in contrast to its apparent minor role in cytoplasmic thiol-redox control, glutathione plays an essential role in mitochondrial thiol-redox regulation.

## 2 Materials and methods

### 2.1 Yeast strains and plasmids

The *S. cerevisiae* strains used in this study were isogenic derivatives of W303 *(MATa ura3-52 leu2-3 leu2-112 trp1-1 ade2-1 his3-11 can1-100).* Strains deleted for the mitochondrial thioredoxin (*trx3::kanMX4*), cytoplasmic thioredoxins (*trx1::TRP1 trx2::URA3*), glutathione reductase (*glr1::TRP1*) and *GSH1* (*gsh1::LEU2*) have been described previously [23], [24]. A mutant version of Glr1 (Glr1^M1L^) was made in *GLR1* with a C-terminal Flag tag contained on plasmid pRS413 or pRS416. The *FET3-lacZ* reporter fusion has been described previously [25].

### 2.2 Growth conditions

Strains were grown in rich YEPD medium (2% w/v glucose, 2% w/v bactopeptone, 1% w/v yeast extract) or minimal SCD medium (0.17% w/v yeast nitrogen base without amino acids, 5% w/v ammonium sulphate, 2% w/v glucose supplemented with appropriate amino acids and bases). For growth on non-fermentable carbon sources, SGE and YEPGE contained 3% (v/v) glycerol and 1% (v/v) ethanol. Media were solidified by the addition of 2% (w/v) agar. Stress sensitivity was determined by growing cells to stationary phase and spotting diluted cultures (OD_600_=1.0, 0.1, 0.01 and 0.001) onto agar plates containing various concentrations of oxidants. Chronological life span experiments were performed in liquid SCD media supplemented with a five-fold excess of uracil, leucine, tryptophan, adenine and histidine to avoid any possible artefacts arising from the auxotrophic deficiencies of the strains.

### 2.3 Redox measurements

The redox state of Trx3 was measured by covalent modification with the thiol-reactive probe 4-acetamido-4′maleimidyldystilbene-2,2′-disulphonic acid (AMS; Molecular Probes) as described previously [26]. Previously described, targeted redox-responsive fluorescent protein probes (roGFP2) were used to quantify cytosolic and mitochondrial matrix redox potentials [27]. To obtain measurements of the fully oxidized (ox) and fully reduced (red) roGFP control probes, cells were treated with 20 mM H_2_O_2_ or 100 mM DTT for 30 min, respectively. Ten thousand cells were counted for each sample using a Becton Dickinson (BD) LSRFortessa™ cell analyser and data were analysed using BD FACSDiva 8.0.1 software. The degree of probe oxidation (OxD) was calculated as described previously [28].

### 2.4 Mitochondrial fractionation and analysis

Cellular fractionation into cytoplasmic and mitochondrial fractions was performed essentially as described by Gregg et al. [29] with minor modifications. Yeast cells were collected by centrifugation and frozen at −80 °C in 1.2 M sorbitol, 50 mM potassium phosphate, pH 7.4. Cells were thawed immediately before use and spheroplasted using 1 mg/g (wet weight) Zymolyase-100T (MP Biomedicals, from *Arthrobacter luteus*) with gentle agitation at room temperature for 30–60 min. Spheroplasting efficiency was determined at regular intervals by measuring the OD_600_ of small aliquots of cells in 1 ml of 0.6% (g/v) SDS. Cell homogenization was achieved using a tissue grinder (glass/Teflon 2 ml, Safe-Grind®, Wheaton). Protein concentrations were measured using a NanoDrop ND-8000 spectrophotometer. Cell fractionation was verified by Western blot analysis using mitochondrial (αPrx1, αPor1) and cytosolic (αPgk1) specific antibodies. Protein extracts were electrophoresed under reducing conditions on SDS-PAGE minigels and electroblotted onto PVDF membrane (Amersham Pharmacia Biotech). Bound antibody was visualized by chemiluminescence (ECL, Amersham Pharmacia Biotech). Lipid peroxidation in mitochondrial fractions was determined using an OxiSelect™ thiobarbituric acid reactive substances assay Kit (TBARS) (Cell Biolabs, Inc.). Methionine oxidation was detected using αMetO antibodies (Novus Biologicals).

### 2.5 β-Galactosidase assays

For the determination of β-galactosidase activity, transformants were assayed essentially as described previously [30]. Cells were grown to early exponential phase and β-galactosidase activity is expressed as nanomoles of o-nitrophenyl-β-d-galactopyranoside (ONPG) hydrolyzed per minute per microgram of total protein (U). Values shown are the means of at least three determinations. The *gsh1* mutant was grown in minimal SCD media in the presence or absence of 1 mM GSH.

### 2.6 Chronological life span

Experiments were performed according to [31]. Briefly, cells were cultured in liquid SCD media (180 rpm, 30 °C) with aliquots taken every 2–3 days for flow cytometry analysis (501 nm excitation, 586 nm/15 nm emission detection on Becton Dickinson (BD) LSRFortessa™ cell analyser, BD FACSDiva 8.0.1 software) after propidium iodide (PI) staining.

### 2.7 Statistical analysis

Data are presented as mean values±SD. Statistical analysis was performed by one-way ANOVA and results were considered statistically significant with a *p*-value less than 0.05.

## 3 Results

### 3.1 A mutant lacking mitochondrial Glr1 is unable to maintain its mitochondrial redox potential

**Fig. 1:**
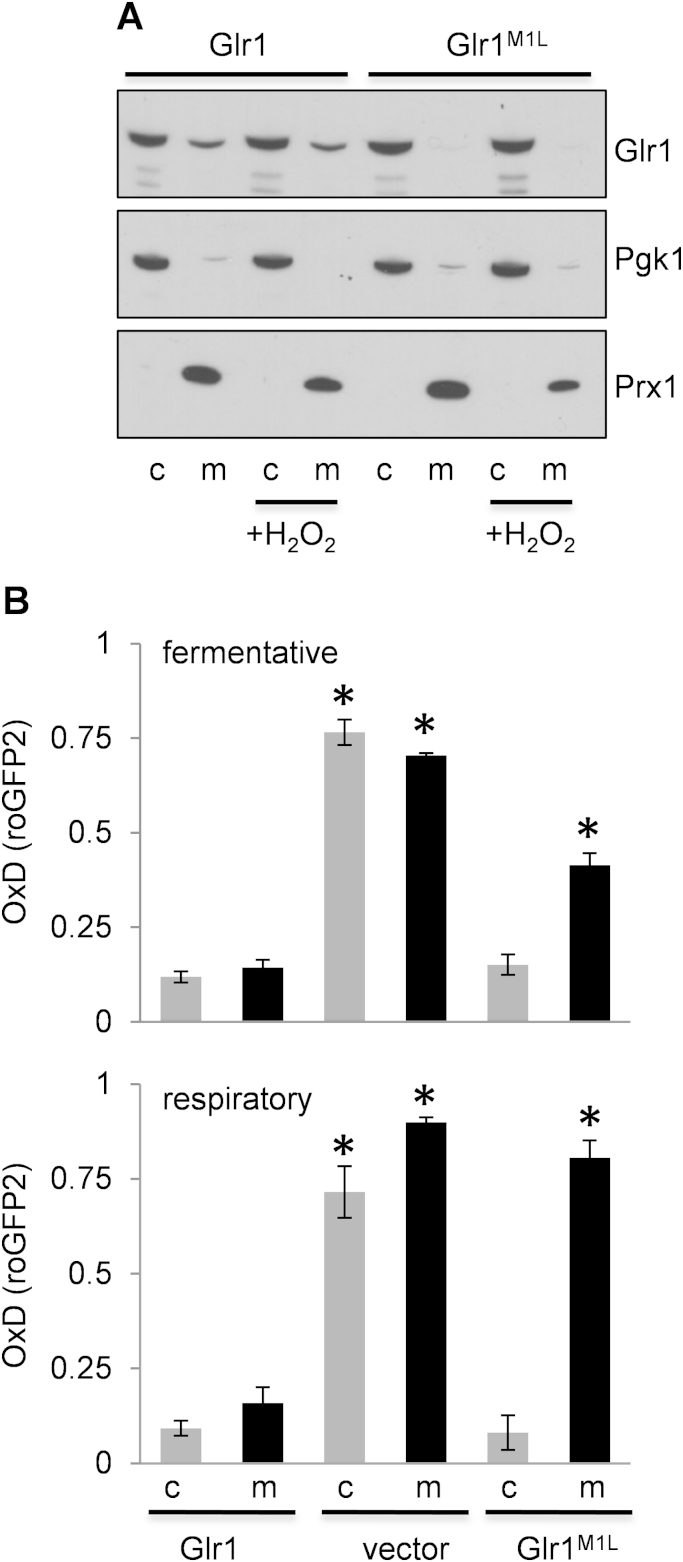
A Glr1^M1L^ mutant is unable to maintain its mitochondrial redox potential. (A) Confirmation of Glr1 localization in *glr1* mutant strains. A *glr1* deletion strain containing Flag-tagged Glr1 or Glr1^M1L^ was grown in SCD media to exponential phase and treated with 1 mM hydrogen peroxide for one hour. Cell lysates were fractionated into cytosolic and mitochondrial fractions and Glr1 localization confirmed by immunoblotting using FLAG antibodies. Antibodies directed against Pgk1 were used as a cytoplasmic marker (c) and against Prx1 as a mitochondrial marker (m). (B) Cytosolic and mitochondrial glutathione redox potentials were measured using targeted roGFP2 fluorescent probes. A *glr1* deletion strain containing empty vector, Glr1 or Glr1^M1L^ was grown to exponential phase under fermentative (SCD media) or respiratory (SGE media) growth conditions. The degree of sensor oxidation (OxD) is shown with an OxD value=1 for the fully oxidized probe and OxD=0 for the fully reduced probe. Data shown are the means of three independent biological repeat experiments±SD. ^⁎^ Indicates a significant difference from the corresponding cytoplasmic or mitochondrial wild-type (Glr1) values (*p*=<0.01).

To examine the mitochondrial requirement for Glr1, we constructed a mutant lacking its first in-frame AUG codon (Glr1^M1L^). The Glr1^M1L^ mutant and wild-type Glr1 were expressed as the sole copies of Glr1 in *glr1* mutant cells. Cells were fractionated and Glr1 detected using western blot analysis (Fig. 1A). This analysis confirmed that wild-type Glr1 localizes to both the cytoplasm and mitochondria as previously described [22]. As expected, the Glr1^M1L^ mutant was detected in the cytoplasm, but not in mitochondria (Fig. 1A). It should be noted that it is not possible to construct a mutant where Glr1 is constitutively localized to mitochondria [22]. Mutation of the second AUG codon (M17L) enriches mitochondrial Glr1, but significant amounts of Glr1 are still present in the cytoplasm, presumably due to overwhelming the mitochondrial import system.

Previously described, targeted roGFP2 fluorescent probes [27] were used to quantify cytosolic and mitochondrial matrix redox potentials in *gl1* mutants. These sensors equilibrate with the local glutathione pool and register thiol redox changes via disulphide bond formation. We calculated the degree of oxidation (OxD) to compare the redox potentials in different intracellular compartments [28], [32]. The cytosolic and mitochondrial matrix OxD values were similar to previously reported values for a wild-type strain grown under fermentative conditions [28]. OxD values were significantly increased in both compartments in the *glr1* mutant as would be expected due to the lack of Glr1 (Fig. 1B, vector alone). Introduction of the Glr1^M1L^ mutant restored the cytosolic OxD value to a similar level as for the wild-type (Fig. 1B). The mitochondrial OxD value was partially restored, but still remained significantly oxidized compared to a strain expressing wild-type Glr1. This was exacerbated under respiratory growth conditions where the oxidation state of mitochondria in the Glr1^M1L^ mutant was comparable to that of a mutant completely lacking Glr1 (Fig. 1B). These data indicate that the Glr1^M1L^ mutant has a similar cytosolic redox potential to that of a wild-type strain, but it is unable to maintain its mitochondrial redox potential. We therefore used this mutant to examine the cellular requirement for the mitochondrial GSSG:2GSH redox couple.

### 3.2 The mitochondrial GSSG:2GSH redox couple is important for cell growth but does not affect iron metabolism

**Fig. 2:**
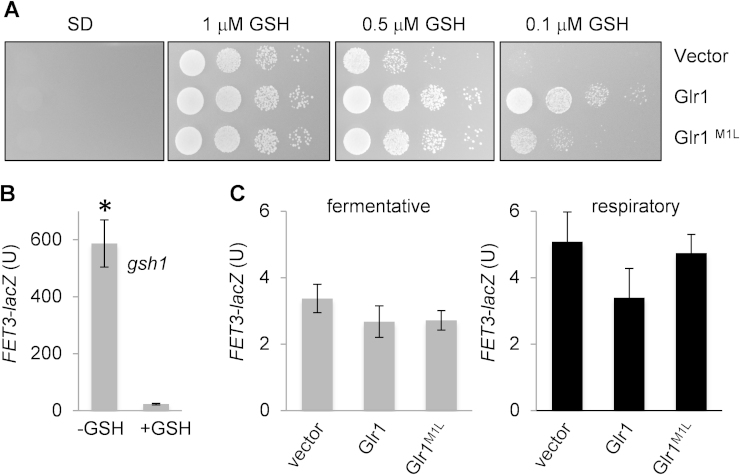
Mitochondrial glutathione is important for cell growth but does not affect iron metabolism. (A) The indicated strains were grown in YEPD media, washed to remove any exogenous GSH, and spotted onto minimal SCD plates containing different concentrations of GSH. Cells were diluted to *A*_600_=1.0, 0.1, 0.01 and 0.001. (B) Glutathione deficiency causes an iron-starvation-like response. A *FET3::LacZ* reporter construct, which is induced by the Aft1 transcription factor in response to iron depletion, was used to detect any alterations in iron homeostasis. *FET3::LacZ* activity was greater than 100-fold higher in a *gsh1* mutant grown in the absence of GSH compared with growth in the presence of exogenous glutathione (+GSH). ^⁎^*p*<0.01. (C) Loss of *GLR1* does not cause an iron-starvation response. No significant differences in *FET3::LacZ* activity were detected in the *glr1* deletion strain containing empty vector, Glr1 or Glr1^M1L^ grown to exponential phase under fermentative (SCD media) or respiratory (SGE media) growth conditions.

We examined a mutant deleted for both *GSH1* and *GLR1* to test whether mitochondrial Glr1 activity is important for maintaining the growth of a *gsh1* mutant. A *gsh1* mutant which cannot make its own GSH, requires exogenous GSH for growth and exhausts its GSH supply when switched to minimal media lacking GSH [6]. Strains were initially grown in liquid cultures containing GSH, before being extensively washed and plated on minimal media plates containing various concentrations of GSH. No growth was observed in the complete absence of GSH and the addition of 1 μM GSH was sufficient to restore the growth of all strains (Fig. 2A). Ten-fold lower concentrations of GSH were sufficient to maintain the growth of a *gsh1 glr1* mutant expressing wild-type Glr1, but not a *gsh1* mutant completely lacking Glr1 activity (Fig. 2A, *gsh1 glr1* vector). These data indicate that the presence of Glr1 improves the growth recovery of a *gsh1* mutant, presumably acting to recycle limiting amounts of oxidized GSSG. The addition of 0.1 μM GSH only partially restored the growth of the *gsh1 glr1* mutant strain containing the Glr1^M1L^ mutant (Fig. 2A), confirming the particular importance of the mitochondrial GSSG:2GSH redox couple for cell growth.

The most prominent phenotype described for mutants depleted of GSH is aberrant iron metabolism due to the requirement for GSH in iron–sulphur cluster assembly [5], [12], [33]. The iron–sulphur cluster assembly machinery is located in the mitochondrial matrix and is required for the maturation of both mitochondrial and cytosolic iron–sulphur proteins [34]. Additional intermembrane space proteins and glutathione are specifically required for the assembly of cytosolic iron–sulphur proteins [12]. It has therefore long been known that cells depleted of GSH are defective in extra-mitochondrial but not mitochondrial iron–sulphur cluster maturation [12], [33]. Defects in iron–sulphur cluster biogenesis cause defects in iron homeostasis including an increase in mitochondrial iron concentrations and a concomitant loss of cytosolic iron. We therefore examined whether the Glr1^M1L^ mutant displays an iron starvation response as a measure of any defect in iron–sulphur cluster biosynthesis. Iron homeostasis in yeast is regulated by the Aft1 transcription factor which activates its target genes in response to low iron conditions [35]. When yeast cells are depleted of GSH, they activate Aft1 which induces Aft1-target genes such as *FET3* [5], [12]. We therefore used a *FET3::LacZ* reporter assay as sensitive measure for any defects in iron homeostasis in glutathione mutants. For comparison, we first examined a *gsh1* mutant grown in minimal media devoid of glutathione. *FET3::LacZ* activity was greater than 100-fold higher in a *gsh1* mutant grown in the absence of GSH compared with growth in the presence of exogenous glutathione (Fig. 2B). In contrast, complete loss of *GLR1* or expression of the Glr1^M1L^ mutant did not significantly increase *FET3::LacZ* activity under fermentative or respiratory conditions compared with a wild-type strain (Fig. 2C). These data indicate that in contrast to glutathione depletion, shifting the GSSG:2GSH redox couple to a more oxidized state does not cause an iron-starvation response.

### 3.3 Mitochondrial Glr1 is required for oxidative stress tolerance

**Fig. 3:**
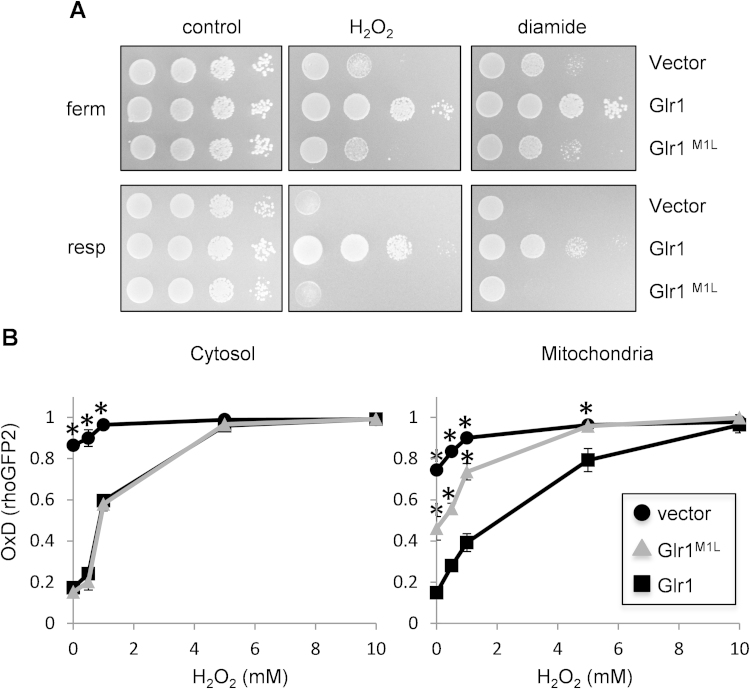
Mutants lacking mitochondrial *GLR1* are sensitive to oxidative stress conditions. (A) Sensitivity to oxidative stress was determined by spotting strains onto minimal media containing various concentrations of hydrogen peroxide or diamide. Results are shown for the *glr1* deletion strain containing empty vector, Glr1 or Glr1^M1L^ following three days growth on 1.2 mM hydrogen peroxide and 0.5 mM diamide. (B) Cytosolic and mitochondrial glutathione pools are oxidized in response to oxidative stress conditions. A *glr1* deletion strain containing empty vector, Glr1 or Glr1^M1L^ was grown to exponential phase under fermentative (SCD media) growth conditions and treated with the indicated concentrations of hydrogen peroxide for one hour. Cytosolic and mitochondrial glutathione redox potentials were measured using targeted roGFP2 fluorescent probes. The degree of sensor oxidation (OxD) is shown with an OxD value=1 for the fully oxidized probe and OxD=0 for the fully reduced probe. Data shown are the means of three independent biological repeat experiments±SD. ^⁎^ Indicates a significant difference from the corresponding cytoplasmic or mitochondrial wild-type (Glr1) values (*p*<0.01).

Mutants lacking *GLR1* are sensitive to oxidative stress conditions induced by exposure to hydrogen peroxide or the thiol oxidant diamide [21], [36]. We therefore examined whether the loss of mitochondrial Glr1 in the Glr1^M1L^ mutant influences oxidant sensitivity. As expected, the *glr1* mutant containing an empty vector was sensitive to hydrogen peroxide compared with a *glr1* mutant complemented with wild-type *GLR1* (Fig. 3A). Unexpectedly however, the Glr1^M1L^ mutant showed a similar sensitivity to hydrogen peroxide as the strain completely lacking *GLR1*. This sensitivity was even more pronounced under respiratory growth conditions (Fig. 3A). Similarly, the *glr1* mutant containing empty vector or Glr1^M1L^ showed a comparable sensitivity to diamide under both fermentative and respiratory growth conditions (Fig. 3A). These findings are surprising because the Glr1^M1L^ strain contains wild-type levels of cytoplasmic Glr1 and its cytoplasmic redox potential is comparable to that of a wild-type strain. This suggests that the loss of mitochondrial Glr1, and hence the ability to maintain the mitochondrial GSSG:2GSH redox couple, specifically accounts for the sensitivity of *glr1* mutants to oxidative stress conditions induced by the addition of an exogenous oxidant.

To examine whether the sensitivity of a Glr1^M1L^ strain to oxidative stress conditions correlates with the redox state of mitochondria, we used targeted roGFP2 fluorescent probes to examine changes in cellular redox potential during oxidative stress conditions. The cytosolic roGFP2 probe was progressively oxidized in the wild type strain in response to treatments with hydrogen peroxide ranging between 0.5 and 10 mM for one hour (Fig. 3B). In comparison, the cytosolic roGFP2 probe underwent an almost identical pattern of oxidation in the Glr1^M1L^ mutant strain (Fig. 3B). These data indicate that cytosolic Glr1^M1L^ is fully functional and cytosolic glutathione is oxidized to a similar extent in strains containing wild-type Glr1 or Glr1^M1L^. The mitochondrial roGFP2 fluorescent probe also became oxidized in a wild-type strain following exposure to hydrogen peroxide confirming that the mitochondrial pool of glutathione is a target of oxidative stress caused by the addition of an exogenous oxidant (Fig. 3B). This oxidation was even more pronounced in the Glr1^M1L^ mutant indicating that the cytosolic GSSG:2GSH redox couple is insufficient to protect mitochondrial glutathione against oxidation during exposure to exogenously added hydrogen peroxide. Both the cytosolic and mitochondrial roGFP2 probes became almost fully oxidized in the *glr1* deletion strain confirming that Glr1 is required to maintain the redox potential of both the cytoplasm and mitochondria during oxidative stress conditions.

We examined the cellular localization of Glr1 to determine whether it changes in response to oxidative stress. However, exposure of cells to oxidative stress induced by hydrogen peroxide did not alter the relative concentrations of Glr1 in cytoplasmic and mitochondrial fractions (Fig. 1A). Similarly, the cytoplasmic localization of Glr1^M1L^ was unaffected by oxidant exposure. Together, these data indicate that the cytosolic glutathione pool is insufficient to protect mitochondrial glutathione against oxidation following exposure to an exogenous oxidant and highlight the importance of mitochondrial glutathione in oxidant tolerance.

### 3.4 Independent redox regulation of the mitochondrial glutathione and thioredoxin systems

**Fig. 4:**
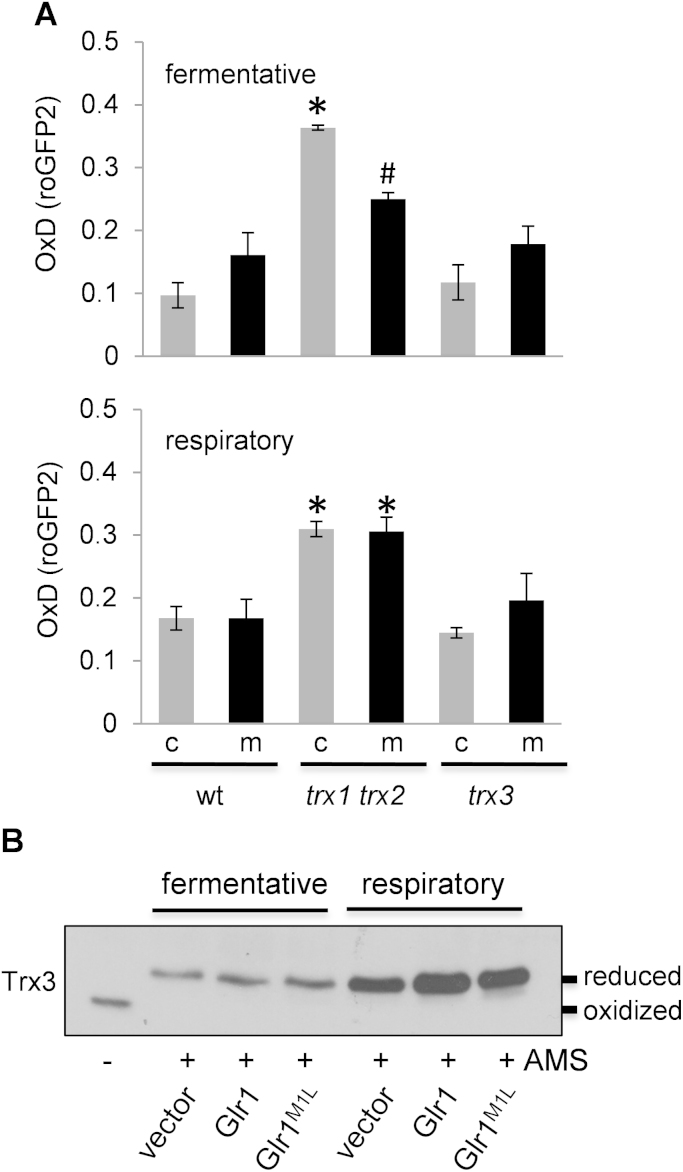
Independent redox regulation of the mitochondrial glutathione and thioredoxin systems. (A) Cytosolic and mitochondrial glutathione redox potentials were measured in mutant strains lacking cytoplasmic thioredoxins (*trx1 trx2*) or mitochondrial thioredoxin (*trx3*). Strains were grown to exponential phase under fermentative (SC media) or respiratory (SGE media) growth conditions. The degree of sensor oxidation (OxD) is shown with an OxD value=1 for the fully oxidized probe and OxD=0 for the fully reduced probe. Data shown are the means of three independent biological repeat experiments±SD. Differences are indicated compared with the corresponding cytoplasmic or mitochondrial wild-type values (^⁎^*p*<0.01, #*p*>0.05). (B) Trx3 does not become oxidized in *glr1* mutants. A *glr1* deletion strain containing empty vector, Glr1 or Glr1^M1L^ was grown to exponential phase under fermentative (SCD media) or respiratory (SGE media) growth conditions. Proteins were precipitated with TCA, and free thiols were modified by reaction with AMS. Samples were separated using SDS-PAGE and mitochondrial Trx3 detected by immunoblotting. Fully oxidized and fully reduced proteins are indicated.

Whole cell glutathione assays have shown that loss of cytoplasmic thioredoxins (*trx1 trx2*) results in an increase in total glutathione levels which is thought to act as a compensatory mechanism in response to the increased redox load [26]. An increase in both GSH and GSSG is detected and the redox ratio (GSH:GSSG) is shifted towards a more oxidized state. We therefore examined whether loss of the mitochondrial thioredoxin (Trx3) influences the mitochondrial glutathione redox state using roGFP2 fluorescent probes. We first examined a mutant lacking cytoplasmic thioredoxins (*trx1 trx2*) and found that OxD values were increased by approximately two-fold for both the cytoplasmic and mitochondrial roGFP2 fluorescent probes under fermentative or respiratory conditions (Fig. 4A). In contrast, OxD values were not increased in the *trx3* mutant indicating that the loss of mitochondrial Trx3 does not impose a redox load on the glutathione system (Fig. 4A).

Loss of *GLR1* has previously been reported to have no effect on the redox state of mitochondrial Trx3 under fermentative or respiratory growth conditions [24]. These studies were performed using a Myc-tagged version of Trx3 and so we repeated this analysis here using native Trx3 probed with a Trx3-specific antibody to ensure that tagging Trx3 does not influence its redox state. Cell extracts were reacted with the thiol-specific probe 4-acetamido-4′maleimidyldystilbene-2,2′-disulfonic acid (AMS) which alkylates Cys residues in a free-SH, but not in an oxidized state. This increases the relative molecular mass of proteins and confirmed that Trx3 is predominantly present in a reduced form in a wild-type strain grown under fermentative or respiratory conditions (Fig. 4B). Loss of *GLR1* (*glr1* vector) or expression of the Glr1^M1L^ mutant did not alter the redox state of Trx3. Taken together, these data indicate that in contrast to the cytoplasmic redox systems, the redox states of mitochondrial glutathione and Trx3 are maintained independently and the mitochondrial GSSG:2GSH redox couple glutathione is unaffected by the loss of the mitochondrial thioredoxin.

### 3.5 Mitochondrial thioredoxin provides a back-up antioxidant system for the glutathione system during oxidative stress conditions

**Fig. 5:**
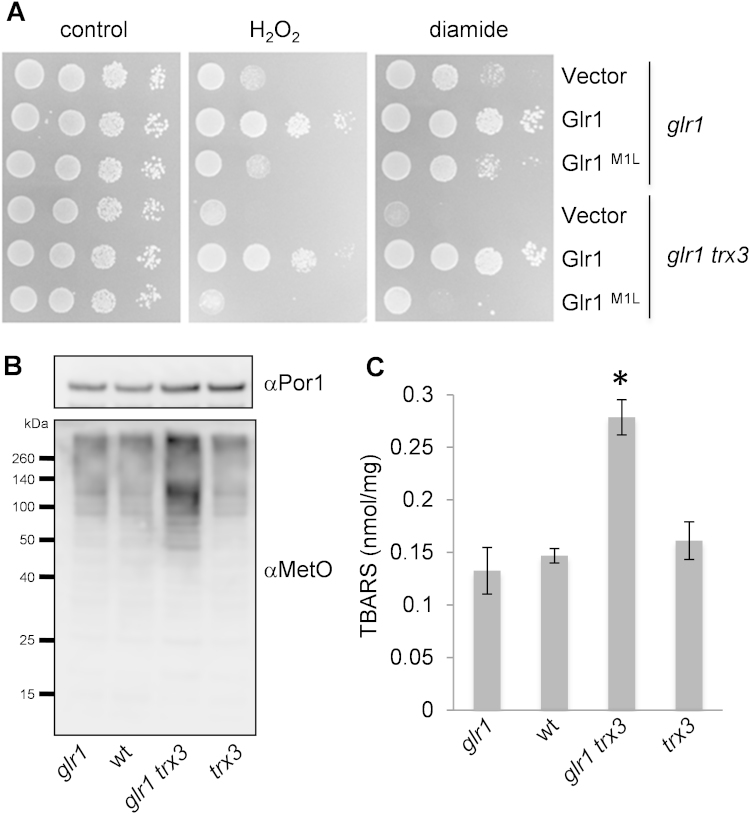
The mitochondrial thioredoxin provides a back-up system for the glutathione system during oxidative stress conditions. (A) Sensitivity to oxidative stress was determined by spotting strains onto SCD media containing various concentrations of hydrogen peroxide or diamide. Results are shown for the *glr1* and *glr1 trx3* deletion strains containing empty vector, Glr1 or Glr1^M1L^ following three days growth on 1.2 mM hydrogen peroxide and 0.5 mM diamide. (B) Methionine oxidation was detected in mitochondrial fractions from the wild-type, *glr1, trx3* and *glr1 trx3* mutant strains using antibodies that recognize methionine sulphoxide (αMetO). Western blots were probed with α-Por1 to confirm that similar amounts of protein were loaded into each lane. (C) Lipid peroxidation was detected in mitochondrial fractions from the wild-type, *glr1, trx3* and *glr1 trx3* mutant strains using an OxiSelect™ TBARS Assay Kit. Data shown are the means of three independent biological repeat experiments±SD. TBARS levels were significantly different in the *glr1 trx3* mutant compared with the wild-type strain (^⁎^*p*<0.01).

Although a complete mitochondrial thioredoxin system comprising a thioredoxin (Trx3) and a thioredoxin reductase (Trr2) has been identified in yeast [37], no function in redox regulation or antioxidant defence has been identified for Trx3 [24], [37]. We have previously shown that the GSSG:2GSH redox couple is required to maintain the redox state of Trx3 during oxidative stress conditions [24]. We therefore constructed mutants lacking *GLR1* and *TRX3* to test whether there is a requirement for Trx3 when the GSSG:2GSH redox couple becomes oxidized. In agreement with previous observations [24], loss of mitochondrial Trx3 does not significantly affect oxidant sensitivity (Fig. 5, compare *glr1 GLR1* with *glr1 trx3 GLR1*). However, loss of Trx3 exacerbated the sensitivity of the *glr1* mutant containing empty vector or Glr1^M1L^ to both hydrogen peroxide and diamide stress. This suggests that Trx3 can act as a back-up antioxidant to promote oxidant tolerance, under conditions where mitochondrial glutathione becomes oxidized.

The possible antioxidant function of Trx3 is currently unknown. Yeast contains a mitochondrial 1-Cys-peroxiredoxin (Prx1) which is active as a peroxidase and can protect cells against hydrogen peroxide stress [38]. However, whilst Trx3 can support the peroxidase activity of Prx1 in vitro [38], [39], Trx3 does not appear to support the antioxidant activity of Prx1 in vivo [39]. We therefore examined markers of oxidative damage in *glr1* and *trx3* mutant strains to confirm whether Trx3 has an antioxidant function. All amino acids are potential targets for oxidation, but methionine residues are particularly sensitive forming methionine sulphoxide (MetO) in cells [40]. We used an anti-MetO antibody to detect methionine oxidation in mitochondrial extracts from *glr1* and *trx3* mutants. We found that the levels of mitochondrial MetO are comparable in wild-type, *glr1* and *trx3* mutants. In contrast, mitochondrial MetO concentrations were elevated in the *glr1 trx3* double mutant (Fig. 5B). Malondialdehyde (MDA) is a reactive aldehyde which is often used as a measure of lipid peroxidation in cells [41]. The levels of mitochondrial MDA are comparable in wild-type, *glr1* and *trx3* mutants, whereas, they were elevated two-fold in the *glr1 trx3* double mutant (Fig. 5C). These data indicate that mitochondrial Trx3 can act in antioxidant defence, but appears to predominantly act as a back-up system for the mitochondrial glutathione system.

### 3.6 The mitochondrial glutathione and thioredoxin systems play an overlapping role in maintaining longevity

**Fig. 6:**
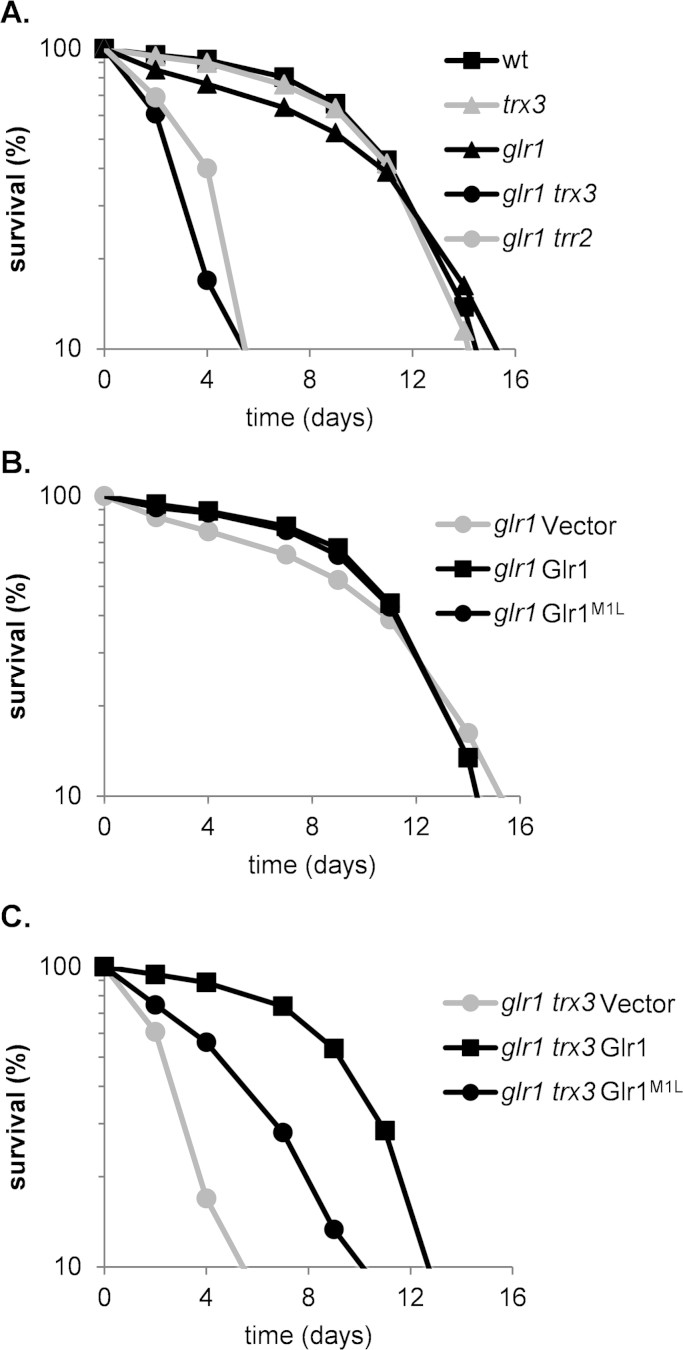
The mitochondrial glutathione and thioredoxin systems play an overlapping role in maintaining longevity. (A–C) Chronological lifespan of the indicated strains was determined by propidium iodide (PI) staining and flow cytometry analysis. Data represent the percentage of live cells in stationary phase cultures relative to day zero.

There are many established links between oxidative stress and ageing, and for example, ROS may play a causal role in cellular decline during ageing [42]. Yeast cells can survive for prolonged periods of time in culture and have been used as a model of the chronological life span (CLS) of mammals, particularly for tissues composed of non-dividing populations. Studies using this model have identified many key conserved ageing factors that modulate ageing [43]. In the CLS model, populations of stationary phase cells are maintained in liquid and viability measured over time. We examined CLS to determine whether the mitochondrial thiol-redox systems are required to maintain longevity. Loss of *GLR1* resulted in a modest decrease in viability during stationary phase, although maximal CLS (~16 days) was unaffected relative to the CLS of wild-type and *trx3* mutant strains (Fig. 6A). In contrast, simultaneous loss of *GLR1* and *TRX3* significantly decreased CLS suggesting that the glutathione and mitochondrial thioredoxin systems are required for maximal longevity (Fig. 6A). CLS appears to require the complete mitochondrial thioredoxin system since a similar reduction in longevity was observed in a mutant lacking both *GLR1* and *TRR2* (Fig. 6A). We further tested the requirement for Glr1 and Trx3 by re-introduction of Glr1 into the *glr1* (Fig. 6B) or *glr1 trx3* (Fig. 6C) mutants. Re-introduction of Glr1 into the *glr1 trx3* mutant restored its pattern of stationary phase survival and CLS comparable to that of the wild-type strain (compare *glr1* Glr1 with *glr1 trx3* Glr1, Fig. 6B and C). However, the Glr1^M1L^ mutant only partially restored stationary phase survival and CLS indicating that the mitochondrial GSSG:2GSH redox couple and thioredoxin systems play an overlapping role in maintaining longevity (Fig. 6C).

## 4 Discussion

Mitochondria are a primary source of ROS in cells. Mitochondrial thiols are therefore major ROS targets and this is exacerbated by the relatively alkaline pH of mitochondria. Hence, redox regulation is critical for numerous mitochondrial functions. For example, GSH deficiency in mammalian cells leads to widespread mitochondrial damage [44] and yeast strains lacking GSH are unable to grow by respiration due to an accumulation of oxidative damage to mitochondrial DNA [8], [45]. Glutathione is synthesized in the cytosol and must be transported into mitochondria via an active energy requiring process [46]. The resulting oxidized GSSG is unable to exit this compartment and is reduced by glutathione reductase [47]. It has been difficult to test the mitochondrial requirement for GSH due to a lack of methodology to specifically deplete mitochondrial GSH. We therefore examined a yeast mutant lacking mitochondrial glutathione reductase to determine the requirement for the mitochondrial GSSG:2GSH redox couple. It has long been known that yeast mutants deleted for *GLR1* accumulate increased levels of GSSG and are sensitive to oxidative stress [21], [36]. We show here that it is the loss of mitochondrial Glr1 which specifically accounts for this sensitivity. Our data indicate that mitochondrial glutathione is crucial for mitochondrial thiol-redox regulation and the mitochondrial thioredoxin system appears to provide a back-up system during oxidative stress conditions, but cannot bear the redox load of the mitochondria on its own.

Previous studies have emphasized the role of glutathione in iron metabolism [5]. However, these studies were performed under conditions of glutathione starvation, a condition which is lethal in cells. Under these conditions, GSH is limiting for iron sulphur cluster formation resulting in an iron starvation response. We did not detect any iron starvation response in a *glr1* mutant using an Aft1-responsive reporter construct, which is strongly induced in response to glutathione depletion. Glutathione depletion is thought to signal iron starvation due to its function in iron sulphur cluster maturation and mitochondrial export [34]. Loss of *GLR1* does not alter the cellular concentration of GSH, but shifts the glutathione redox ratio (GSH:GSSG) to a more oxidized state [36], [48]. Previous studies have shown that the steady state concentration of mitochondrial glutathione is approximately 20 nmol/mg protein compared with 100–200 nmol/mg protein in the cytosol [22], [39]. Importantly, the amounts of mitochondrial and cytosolic glutathione (GSH+GSSG) are similar in the wild-type and *glr1* mutant strains [22]. Whilst total glutathione levels are unaffected, the *glr1* mutant has a much greater percentage of oxidized glutathione in both the cytosol and the mitochondria compared with a wild-type strain. Hence, there appears to be sufficient GSH available in a *glr1* mutant to maintain iron sulphur cluster assembly, which is proposed to only require trace amounts [5]. Despite not affecting iron metabolism, mitochondrial glutathione is particularly important for yeast growth since higher concentrations of glutathione are required to rescue a *gsh1 Glr1*^*M1L*^ mutant compared with a *gsh1 GLR1* mutant. This emphasizes the particular importance of the mitochondrial GSSG:2GSH redox couple for cell growth and oxidant tolerance, since the cytoplasmic GSSG:2GSH redox couple is maintained at wild-type levels in the Glr1^M1L^ mutant.

Transport of hydrogen peroxide across cell membranes can be facilitated by transporters such as aquaporins, but once inside cells it should be freely diffusible [49]. Hydrogen peroxide causes oxidative stress, but also plays important roles as a signalling molecule in the regulation of many biological processes [50]. We found that the cytosolic roGFP2 probe was similarly oxidized in response to hydrogen peroxide addition in both the wild-type and Glr^M1L^ mutant suggesting that similar cytosolic redox environments are maintained in these strains. The fact that the mitochondrial roGFP2 probe becomes more oxidized in a mutant lacking mitochondrial Glr1 suggests that the cytosolic pool of glutathione is insufficient to protect mitochondria against oxidation following exposure to an exogenous oxidant. It is unclear whether oxidant sensitivity and mitochondrial glutathione oxidation in a *glr1* mutant is caused by loss of an antioxidant activity which is normally mediated by glutathione. For example, mitochondrial glutaredoxin (Grx2) and peroxiredoxin (Prx1) require glutathione for their antioxidant activities [39], [51], [52]. Glutathione oxidation may also reflect the activity of glutathione itself as an antioxidant. Glutathione has been suggested to act as an antioxidant which can directly scavenge ROS such as the hydroxyl radical [53], [54], although this has been challenged and the redox function of glutathione may require enzymes [55]. Alternatively, the oxidant sensitivity of a *glr1* mutant might arise due to a general loss of mitochondrial thiol redox regulation, and non-specific oxidation of reactive cysteine residues in mitochondrial proteins.

Extensive overlaps have been reported between the yeast thioredoxin and glutathione systems [13], [56]. For example, increased glutathione levels are observed in the absence of cytoplasmic Trx1 and Trx2 which is thought to act as a compensatory mechanism in response to the increased redox load in this mutant [26]. Both GSH and GSSG are increased, shifting the cellular glutathione redox balance to a more oxidized state. These studies were performed using whole cell extracts, rather than compartment-specific measurements. We found here that both the cytoplasmic and mitochondrial roGFP2 fluorescent probes are more oxidized in a *trx1 trx2* mutant, whereas there is no significant alteration in a *trx3* mutant. It is surprising that the mitochondrial glutathione pool is oxidized in a *trx1 trx2* mutant since previous studies using GFP-based redox sensors have suggested that the glutathione pools in the cytosol and mitochondrial matrix are not linked [20], [28]. Hence there must be some previously unrecognized cross-talk between the cytoplasmic thioredoxin system and the mitochondrial glutathione system. Conversely, shifting the glutathione pool to a more oxidized form in the *glr1* mutant does not affect the redox state of cytoplasmic thioredoxins (Trx1, Tx2), and thioredoxin oxidation is only detected in response to severe glutathione depletion [26], [57]. This independent redox regulation of the cytoplasmic thioredoxins is thought to ensure the survival of cells under conditions where the cytoplasmic pool of glutathione is oxidized [26].

Loss of mitochondrial *TRX3* does not alter the redox state of the cytosolic or mitochondrial roGFP2 fluorescent probes suggesting that loss of the mitochondrial thioredoxin does not impose a redox load on the glutathione system. Mitochondrial thioredoxin is however, known to be more reliant on the glutathione redox couple. Unlike cytoplasmic thioredoxins, the redox state of mitochondrial Trx3 is buffered by the GSSG:2GSH redox couple under oxidative stress conditions [24]. Oxidized Trx3 is not detected in a mutant lacking thioredoxin reductase (*trr2*), but is accumulated in a *trr2 glr1* double mutant under both fermentative and respiratory growth conditions [24]. This suggests that mitochondrial thioredoxin function can be regulated by the glutathione system. No cellular requirement for the yeast mitochondrial Trx3 has previously been reported since it is dispensable for growth under normal and oxidative stress conditions [37]. This is surprising since mammalian mitochondrial Trx2 is required for normal development of the mouse embryo and lack of Trx2 results in embryonic lethality [58]. Our current data indicate that Trx3 is required as an antioxidant in the absence of *GLR1,* since the oxidant sensitivity of a *glr1* mutant is exacerbated in the absence of *TRX3* and markers of oxidant damage including MetO and MDA accumulate in a *glr1 trx3* double mutant. We therefore suggest that mitochondrial Trx3 plays an ancillary or back-up role for mitochondrial glutathione, perhaps under stress or growth conditions where mitochondrial glutathione is oxidized.

It was originally reported that Trx3 is the physiological electron donor for mitochondrial Prx1, which is unexpected since 1-Cys Prx’s are not thought to form a disulphide which could act as a substrate for thioredoxin [38]. In vitro assays have confirmed that Trx3 and Trr2 can support Prx1 activity with hydrogen peroxide as a substrate suggesting that Trx3 can directly reduce the sulphenic acid form of the Prx1 peroxidatic cysteine residue [39]. Similarly, recent evidence has suggested that Trx3 can deglutathionylate a mixed disulphide formed between Prx1 and GSH [52]. Although Trx3 does not appear to support Prx1 activity in vivo, it may therefore function to reduce the sulphenic acid or mixed disulphide intermediates formed in other thiol containing proteins. This does not appear to be a major function in oxidative stress defence since our current data indicate that Trx3 is only required for oxidant tolerance in the absence of mitochondrial Glr1. Interestingly, our data indicate that mitochondrial glutathione and Trx3 appear to play a redundant role in longevity. Loss of *GLR1* or *TRX3* alone does not significantly alter chronological lifespan. However, in a *glr1 trx3* double mutant longevity is significantly reduced. The role of Trx3 in CLS appears to require a complete mitochondrial thioredoxin system since a similar defect in CLS is observed in a *glr1 trr2* mutant. This is the first time a thioredoxin system-dependent phenotype has been described for Trx3 since the only other reported phenotypes have been detected in a *trr2* mutant and not a *trx3* mutant [24], [37].

## 5 Conclusions

Thiol redox regulation plays a long-recognized role in the response of cells to oxidative stress conditions. Our current data emphasize the importance of compartmentalized redox regulation when cells are subjected to oxidative stress conditions. Whilst cytosolic glutathione represents the first major pool of thiols which would be a target of oxidation in response to exposure to an exogenous oxidant, it is the mitochondrial glutathione pool which is crucial for oxidant tolerance.

## References

[1] Shi Z.Z., Osei-Frimpong J., Kala G., Kala S.V., Barrios R.J., Habib G.M., Lukin D.J., Danney C.M., Matzuk M.M., Lieberman M.W. (2000). Glutathione synthesis is essential for mouse development but not for cell growth in culture. Proc. Natl. Acad. Sci. USA.

[2] Jain A., Martensson J., Einar E., Auld P.A.M., Meister A. (1991). Glutathione deficiency leads to mitochondrial damage in brain. Proc. Natl. Acad. Sci. USA.

[3] Meister A., Anderson M.E. (1983). Glutathione. Ann. Rev. Biochem..

[4] Schafer F.Q., Buettner G.R. (2001). Redox environment of the cell as viewed through the redox state of the glutathione disulfide/glutathione couple. Free Radic. Biol. Med..

[5] Kumar C., Igbaria A., D’Autreaux B., Planson A.G., Junot C., Godat E., Bachhawat A.K., Delaunay-Moisan A., Toledano M.B. (2011). Glutathione revisited: a vital function in iron metabolism and ancillary role in thiol-redox control. Embo J..

[6] Grant C.M., MacIver F.H., Dawes I.W. (1996). Glutathione is an essential metabolite required for resistance to oxidative stress in the yeast *Saccharomyces cerevisiae*. Curr. Genet..

[7] Wu A., Moye-Rowley W.S. (1994). *GSH1*, which encodes γ-glutamylcysteine synthetase, is a target gene for yAP-1 transcriptional regulation. Mol. Cell. Biol..

[8] Lee J.-C., Straffon M.J., Jang T.-Y., Grant C.M., Dawes I.W. (2001). The essential and ancillary role of glutathione in *Saccharomyces cerevisiae*: Studies with a grande *gsh1* disruptant strain. FEMS Yeast Res..

[9] Spector D., Labarre J., Toledano M.B. (2001). A genetic investigation of the role of glutathione: mutations in the proline biosynthesis pathway are the only suppressors of glutathione auxotrophy in yeast. J. Biol. Chem..

[10] Camier S., Ma E., Leroy C., Pruvost A., Toledano M., Marsolier-Kergoat M.C. (2007). Visualization of ribonucleotide reductase catalytic oxidation establishes thioredoxins as its major reductants in yeast. Free Radic. Biol. Med..

[11] Kispal G., Csere P., Guiard B., Lill R. (1997). The ABC transporter Atm1p is required for mitochondrial iron homeostasis. FEBS Lett..

[12] Sipos K., Lange H., Fekete Z., Ullmann P., Lill R., Kispal G. (2002). Maturation of cytosolic iron–sulfur proteins requires glutathione. J. Biol. Chem..

[13] Toledano M.B., Delaunay-Moisan A., Outten C.E., Igbaria A. (2013). Functions and cellular compartmentation of the thioredoxin and glutathione pathways in yeast. Antioxid. Redox Signal..

[14] Stephen D.W., Jamieson D.J. (1996). Glutathione is an important antioxidant molecule in the yeast *Saccharomyces cerevisiae*. FEMS Microbiol. Lett..

[15] Michiels C., Raes M., Toussaint O., Remacle J. (1994). Importance of Se-glutathione peroxidase, catalase, and Cu/Zn-SOD for cell survival against oxidative stress. Free Radic. Biol. Med..

[16] Ohdate T., Kita K., Inoue Y. (2010). Kinetics and redox regulation of Gpx1, an atypical 2-Cys peroxiredoxin, in *Saccharomyces cerevisiae*. FEMS Yeast Res..

[17] Tanaka T., Izawa S., Inoue Y. (2005). GPX2, encoding a phospholipid hydroperoxide glutathione peroxidase homologue, codes for an atypical 2-Cys peroxiredoxin in Saccharomyces cerevisiae. J. Biol. Chem..

[18] Delaunay A., Pflieger D., Barrault M.B., Vinh J., Toledano M.B. (2002). A thiol peroxidase is an H_2_O_2_ receptor and redox-transducer in gene activation. Cell.

[19] Chance B., Sies H., Boveris A. (1979). Hydroperoxide metabolism in mammalian organs. Physiol. Rev..

[20] Hu J., Dong L., Outten C.E. (2008). The redox environment in the mitochondrial intermembrane space is maintained separately from the cytosol and matrix. J. Biol. Chem..

[21] Grant C.M., Collinson L.P., Roe J.-H., Dawes I.W. (1996). Yeast glutathione reductase is required for protection against oxidative stress and is a target gene for yAP-1 transcriptional regulation. Mol. Microbiol..

[22] Outten C.E., Culotta V.C. (2004). Alternative start sites in the *S. cerevisiae* GLR1 gene are responsible for mitochondrial and cytosolic isoforms of glutathione reductase. J. Biol. Chem..

[23] Trotter E.W., Grant C.M. (2002). Thioredoxins are required for protection againts a reductive stress in the yeast *Saccharomyces cerevisiae*. Mol. Microbiol..

[24] Trotter E.W., Grant C.M. (2005). Overlapping roles of the cytoplasmic and mitochondrial redoc regulatory systems in the yeast *Saccharomyces cerevisiae*. Eukaryot. Cell.

[25] Blaiseau P.L., Lesuisse E., Camadro J.M. (2001). Aft2p, a novel iron-regulated transcription activator that modulates, with Aft1p, intracellular iron use and resistance to oxidative stress in yeast. J. Biol. Chem..

[26] Trotter E.W., Grant C.M. (2003). Non-reciprocal regulation of the redox state of the glutathione/glutaredoxin and thioredoxin systems. Embo Rep..

[27] Ayer A., Fellermeier S., Fife C., Li S.S., Smits G., Meyer A.J., Dawes I.W., Perrone G.G. (2012). A genome-wide screen in yeast identifies specific oxidative stress genes required for the maintenance of sub-cellular redox homeostasis. PLoS One.

[28] Kojer K., Bien M., Gangel H., Morgan B., Dick T.P., Riemer J. (2012). Glutathione redox potential in the mitochondrial intermembrane space is linked to the cytosol and impacts the Mia40 redox state. Embo J..

[29] C. Gregg, P. Kyryakov, V.I. Titorenko, Purification of mitochondria from yeast cells, J. Vis. Exp., 2009. 10.3791/1417 PMC3149909 19704406

[30] Rose M., Botstein D. (1983). Construction and use of gene fusions to *lacZ* (β-galactosidase) which are expressed in yeast. Methods Enzymol..

[31] Ocampo A., Barrientos A. (2011). Quick and reliable assessment of chronological life span in yeast cell populations by flow cytometry. Mech. Ageing Dev..

[32] Morgan B., Sobotta M.C., Dick T.P. (2011). Measuring E(GSH) and H_2_O_2_ with roGFP2-based redox probes. Free Radic. Biol. Med..

[33] Rutherford J.C., Jaron S., Winge D.R. (2003). Aft1p and Aft2p mediate iron-responsive gene expression in yeast through related promoter elements. J. Biol. Chem..

[34] Lill R. (2009). Function and biogenesis of iron–sulphur proteins. Nature.

[35] Yamaguchi-Iwai Y., Stearman R., Dancis A., Klausner R.D. (1996). Iron-regulated DNA binding by the AFT1 protein controls the iron regulon in yeast. Embo J..

[36] Muller E.G.D. (1996). A glutathione reductase mutant of yeast accumulates high levels of oxidized glutathione and requires thioredoxin for growth. Mol. Biol. Cell.

[37] Pedrajas J.R., Kosmidou E., Miranda-Vizuete A., Gustafsson J.-A., Wright A.P.H., Spyrou G. (1999). Identification and functional characterization of a novel mitochondrial thioredoxin system in *Saccharomyces cerevisiae*. J. Biol. Chem..

[38] Pedrajas J.R., Miranda-Vizuete A., Javanmardy N., Gustafsson J.-A., Spyrou G. (2000). Mitochondria of *Saccharomyces cerevisiae* contain one-conserved cysteine type peroxiredoxin with thioredoxin peroxidase activity. J. Biol. Chem..

[39] Greetham D., Grant C.M. (2009). Antioxidant activity of the yeast mitochondrial 1-Cys peroxiredoxin is dependent on thioredoxin reductase and glutathione in vivo. Mol. Cell. Biol..

[40] Stadtman E.R., Levine R.L. (2003). Free radical-mediated oxidation of free amino acids and amino acid residues in proteins. Amino Acids.

[41] Esterbauer H. (1993). Cytotoxicity and genotoxicity of lipid-oxidation products. Am. J. Clin. Nutr..

[42] Harman D. (1972). The biologic clock: the mitochondria?. J. Am. Geriatr. Soc..

[43] Kaeberlein M. (2010). Lessons on longevity from budding yeast. Nature.

[44] Meister A. (1995). Mitochondrial changes associated with glutathione deficiency. Biochem. Biophys. Acta.

[45] Ayer A., Tan S.X., Grant C.M., Meyer A.J., Dawes I.W., Perrone G.G. (2010). The critical role of glutathione in maintenance of the mitochondrial genome. Free Radic. Biol. Med..

[46] Griffith O.W., Meister A. (1985). Origin and turnover of mitochondrial glutathione. Proc. Natl. Acad. Sci. USA.

[47] Olafsdottir K., Reed D.J. (1988). Retention of oxidized glutathione by isolated rat liver mitochondria during hydroperoxide treatment. Biochim. Biophys. Acta.

[48] Grant C.M., MacIver F.M., Dawes I.W. (1996). Stationary phase induction of *GLR1* expression is mediated by the yAP-1 transcriptional protein in *Saccharomyces cerevisiae*. Mol. Microbiol..

[49] Bienert G.P., Moller A.L., Kristiansen K.A., Schulz A., Moller I.M., Schjoerring J.K., Jahn T.P. (2007). Specific aquaporins facilitate the diffusion of hydrogen peroxide across membranes. J. Biol. Chem..

[50] Veal E.A., Day A.M., Morgan B.A. (2007). Hydrogen peroxide sensing and signaling. Mol. Cell.

[51] Luikenhuis S., Dawes I.W., Grant C.M. (1997). The yeast *Saccharomyces cerevisiae* contains two glutaredoxin genes that are required for protection against reactive oxygen species. Mol. Biol. Cell.

[52] J.R.Pedrajas, B. McDonagh, F. Hernandez-Torres, A. Miranda-Vizuete, R. Gonzalez-Ojeda, E. Martinez-Galisteo, C.A. Padilla, J.A. Barcena, Glutathione is the resolving thiol for thioredoxin peroxidase activity of 1-Cys peroxiredoxin without being consumed during the catalytic cycle, Antioxid. Redox Signal., 2015. 10.1089/ars.2015.6366 26159064

[53] Saez G.T., Valls V., Muniz P., Perez-Broseta C., Iradi A., Oliva M.R., Bannister J.V., Bannister W.H. (1993). The role of glutathione in protection against DNA damage induced by rifamycin SV and copper(II) ions. Free Radic. Res. Commun..

[54] Muniz P., Valls V., Perez-Broseta C., Iradi A., Climent J.V., Oliva M.R., Saez G.T. (1995). The role of 8-hydroxy-2′-deoxyguanosine in rifamycin-induced DNA damage. Free Radic. Biol. Med..

[55] Berndt C., Lillig C.H., Flohe L. (2014). Redox regulation by glutathione needs enzymes. Front. Pharmacol..

[56] Morano K.A., Grant C.M., Moye-Rowley W.S. (2011). The response to heat shock and oxidative stress in *Saccharomyces cerevisiae*. Genetics.

[57] Wheeler G.L., Trotter E.W., Dawes I.W., Grant C.M. (2003). Coupling of the transcriptional regulation of glutathione biosynthesis to the availability of glutathione and methionine via the Met4 and Yap1 transcription factors. J. Biol. Chem..

[58] Nonn L., Williams R.R., Erickson R.P., Powis G. (2003). The absence of mitochondrial thioredoxin 2 causes massive apoptosis, exencephaly, and early embryonic lethality in homozygous mice. Mol. Cell. Biol..

